# Potato Starch Hydrogels Produced by High Hydrostatic Pressure (HHP): A First Approach

**DOI:** 10.3390/polym11101673

**Published:** 2019-10-14

**Authors:** Dominique Larrea-Wachtendorff, Gipsy Tabilo-Munizaga, Giovanna Ferrari

**Affiliations:** 1 Department of Industrial Engineering, University of Salerno, 84084 Fisciano (SA), Italy; gferrari@unisa.it; 2 Food Engineering Department, University of Bio Bio, Chillán 3780000, Chile; gtabilo@ubiobio.cl; 3 ProdAl Scarl, Competence Center on Agro-Food Productions, University of Salerno, 84084 Fisciano (SA), Italy

**Keywords:** high hydrostatic pressure, hydrogels, potato starch, gelatinization, FTIR, rheology, gel formation

## Abstract

Starch-based hydrogels have received considerable interest due to their safe nature, biodegradability and biocompatibility. The aim of this study was to verify the possibility of producing natural hydrogels based on potato starch by high hydrostatic pressure (HHP), identifying suitable processing conditions allowing to obtain stable hydrogels, as well as to characterize structural and mechanical properties of these products. Sieved (small size granules and medium size granules) and unsieved potato starch samples were used to prepare aqueous suspensions of different concentrations (10–30% w/w) which were processed at 600 MPa for 15 min at different temperatures (25, 40 and 50 °C). Products obtained were characterized by different techniques (light and polarized microscopy, Fourier transform infrared spectroscopy (FTIR), rheology and differential scanning calorimetry (DSC)). Results obtained so far demonstrated that potato starch suspensions (20% starch–water concentration (w/w)) with granules mean size smaller than 25 µm treated at 600 MPa for 15 min and 50 °C showed a complete gelatinization and gel-like appearance. Potato HHP hydrogels were characterized by high viscosity, shear-thinning behavior and a highly structured profile (G’ >> G’’). Moreover, their FTIR spectra, similarly to FTIR profiles of thermal gels, presented three absorption bands in the characteristic starch-gel region (950–1200 cm^−1^), whose intensity increased with decreasing the particle size and increasing the processing temperature. In conclusion, potato starch hydrogels produced by HHP in well-defined processing conditions exhibited excellent mechanical properties, which can be tailored according to the requirements of the different applications envisaged.

## 1. Introduction

Hydrogels represent a group of materials, composed of three-dimensional crosslinked networks of hydrophilic/hydrophobic polymers, capable to absorb and retain a significant amount of water [1].

They have been listed as “smart structures” whose tailor-made design confers them different functional attributes to be used in the design, synthesis and self-assembly of novel biomaterials and drug delivery systems [2,3]. Since their first application in the production of contact lenses, reported by Wichterle and Lim (1960), currently hydrogels are widely used in several applications such as tissue engineering, controlled drug delivery, agriculture, bioactives protection and water purification, among others [3,4,5,6,7,8].

The production of hydrogels using natural or synthetic polymers has been extensively investigated and, in general, involves intermolecular interactions, covalent and non-covalent interactions induced by physical or chemical treatments, being the crosslinking method and the graft polymerization the most traditional processes [9,10,11]. Hydrogels produced from natural ingredients such as alginate, gelatine, cellulose, chitosan and starch [1,12,13,14,15,16,17,18,19,20], have received increasing attention in the last years, due to their safety, biocompatibility and biodegradability [21]. The utilization of starches has been receiving increasing attention due to the abundance, availability, cost-effectiveness, manageability, high swelling capacity and the versatility of these kinds of materials [21]. Rice, potato, corn and other soluble starches, in fact, have been successfully utilized to produce starch-based hydrogels [1,22,23,24].

In general, the production of starch-based hydrogels involves chemical cross-linking or physical gelatinization to form a stable three-dimensional network [1]. By heating a water suspension with a certain starch concentration, gelatinization occurs, being the main structural change occurring to starch granules [25]. Structural changes take place at defined process conditions, according to the characteristics of the starch source [26]. Gelatinization is a phase transition process from an ordered to a disordered status [27]. Initially, fast swelling of starch granules is observed, due to particles hydration, while during heating amylose leaching and granules disintegration are likely to occur. Finally, during the retrogradation stage, hydrogels form due to the recrystallization and reorganization of the polysaccharide structure [21]. Several techniques, including differential scanning calorimetry (DSC), Fourier transform infrared spectroscopy (FTIR), optical microscopy and rheology have been used to detect phase transition, structural changes and mechanical characteristics of starches during and after the gelatinization step [25,28,29,30].

Starch gelatinization can be also achieved by applying innovative technologies, such as high hydrostatic pressure (HHP), at more suitable processing conditions than those of traditional methods [31,32]. HHP, the most diffused non-thermal technology at the industrial level, is mainly used for microbial inactivation in liquid, solid and particulate foods. It is well known that HHP causes the disordering of biopolymers, including proteins and starches, due to the modifications of non-covalent intermolecular interactions such as those inducing pressure-assisted gelatinization [33]. Under high pressure, starches undergo morphological and structural changes, exhibiting different gelatinization extent, with intact granules and limited swelling of starch particles remaining after treatment, and different rheology compared to thermally treated starches [34,35,36]. In the last 20 years, many studies have been carried out to evaluate the modifications and gelatinization of starches from different plant sources induced by HHP [37]. It has been shown that the effects of HHP on starches depend on the type of starch, water concentration in the suspension and processing conditions, namely pressure level, temperature and processing time [38,39]. It has been observed that, at room temperature, HHP treatments at 600 MPa cause the complete gelatinization of starches (wheat, rice, beans, corn, quinoa and tapioca) in aqueous suspensions [32,36,40,41,42,43,44]. As far as potato starch is concerned, it has been demonstrated that this starch shows a high resistance to pressure-induced gelation, thus very high-pressure levels are required to achieve the complete gelatinization of potato starch–water suspensions [32,45,46]. However, playing potato starch has a very important role in the food industry as a coating, blending, bulking and thickening agent and, more recently, as a gel-hardener, a deeper understanding of its behavior during HHP assisted gelation would be of utmost importance for future industrial application of HHP hydrogels [47]. Muhr and Blanshard (1982), proved that to obtain the complete gelatinization of potato starch aqueous suspensions (12.8% w/w) at 23 °C pressures of about 800 MPa were needed [48]. Bauer and Knorr (2005) achieved the complete gelatinization of 5% (w/w) potato starch aqueous suspensions at processing conditions of 700 MPa for 15 min only by applying a simultaneous thermal treatment at moderate temperature (50 °C) [39]. Other authors demonstrated that HHP treatments of potato starch suspensions at 1000 MPa and different treatment times were effective to achieve gelation only with holding times well above one hour [45,46].

To the best of our knowledge, while many efforts have been devoted to understanding and achieving the HHP gelatinization of potato starch, no studies on the evaluation of structural and mechanical properties of the hydrogels obtained at different processing conditions have been reported in the literature.

Thus, the aim of this study was verifying the possibility of producing potato starch HHP-hydrogels, identifying the most suitable processing conditions to obtain stable hydrogels, and determine structural and mechanical properties of the products obtained.

This investigation provides information allowing to forecast future further applications of these kinds of materials.

## 2. Materials and Methods

### 2.1. Materials

Potato starch powder (S4251, CAS: 9005-25-8, Sigma Aldrich, Steinheim, Germany) was utilized in this experimental work. Before its use, it was characterized by chemical and physical measurements. Potato starch composition was 13.5% moisture content (determined according to the method 925.10, AOAC, 2005), 0.8% fat (determined according to the method 922.06, AOAC, 2005), 0.3% ash (determined according to the method 923.03, AOAC, 2005) and 25% amylose (determined by Megazyme amylose/amylopectin assay kit, Megazyme International Ireland Ltd., Wicklow, Ireland).

Potato starch particle size distribution was evaluated by dynamic light scattering (DLS), using a Malvern Mastersizer 2000 instrument (Malvern Instruments Ltd., Worcestershire, UK). A monomodal distribution was determined with a D [4.3] of 48.75 µm, and a volumetric distributions of 3% of d(0.1) = 19.53 µm, 10.5% of d(0.5) = 45.52 µm and 5.66% of d(0.9) = 89.81 µm.

### 2.2. Experimental Protocol

In this investigation, the effects of solution formulation and processing conditions to obtain potato starch-based hydrogels by HHP were studied through a cascade methodology approach. 

#### 2.2.1. Samples Preparation

Potato starch was suspended in distilled water at different concentrations (10%, 20% and 30% (w/w)) and the solution was formed under gentle mixing. To avoid particles settling, the solutions were prepared immediately before HHP-treatments.

In order to test the effects of PSD on HHP hydrogels formation, samples of potato starch with different mean particle size were separated utilizing laboratory scale sieves with different openings (Endecotts LTD, London, England) coupled to a multidimensional high performance sieving machine (Pbi international, IG/3/Export, Milano, Italy). Sieving was carried out on samples of native potato starch of 15 g each and sieving time was set at 2 min. The different fractions from sieving were collected as function of the particles diameter (dp) as follows: dp < 25 µm, 25 µm < dp < 36 µm, 36 µm < dp < 50 µm, 50 µm < dp < 63 µm, 63 µm < dp < 90 µm and dp > 90 µm. Only two fractions of potato starch were utilized for HHP hydrogel preparation: Medium granules (36 µm < dp < 50 µm) and small granules (dp < 25 µm), with weight fractions of 41% and 7%, respectively. 

For the sake of comparison, also a sample of unsieved potato starch was used in the experiments. Each potato starch fraction was suspended in distilled water at 20% w/w and immediately subjected to HHP-treatments.

To evaluate the effects of combined HHP and thermal treatments at moderate temperature on hydrogel formation only potato starch suspensions (20% w/w) with small granule sizes (<25 µm) were used.

#### 2.2.2. HHP Treatments

For each test, 3 mL of starch suspension was introduced in a flexible bag made of a polymer/aluminum/polymer film (OPP30-A19-LDPE70), deaerated and sealed. Plastic bags were thoroughly mixed and introduced in the high pressure multivessel unit U-111 (UNIPRESS-Polish Academy of Sciences, Warsaw, Poland) equipped with a thermostatic bath (Huber CC245 wl, Offenburg, Germany). A silicon oil (Huber thermofluid M40.165/220.10, Offenburg, Germany) was used as a pressurizing medium as well as the main fluid of the thermostatic bath. The HP unit can be operated at pressures up to 700 MPa in the temperature range from −40 to 100 °C. The high pressure vessels, with an inner volume of 9 mL, were made of a highly conductive material (Cu–Be alloy), which ensures the internal equilibration of temperature during HP treatment [49].

In this investigation, based on our experimental results (data not shown) and previous observations reported by Rubens et al. [50], we utilized a pressure level of 600 MPa for 15 min, commonly used in industrial productions, which are suitable for an economically attractive industrial process, being the challenge of this investigation the successful production of potato starch hydrogels with the right formulation.

Pressurization and decompression rates were set at 8 MPa/s, while the temperature of the pressure vessel was set at 25 °C for all the experiments carried out to evaluate the effects of water concentration in the starch solution and particle size distribution on gel formation, while in combined HP-thermal treatments the temperature of the vessels was set at 40 °C and 50 °C. Temperature oscillation was always ±2 °C. In all cases to reach the set temperature, plastic bags containing the samples were kept in the vessel for 5 min before starting the compression phase.

A rheometer TA instruments AR 2000 (TA instruments, New Castle, DE, USA) equipped with a starch cell and a starch pasting rotor (545,751.001) was used to produce gels by thermal treatments. The following processing parameters were used: Step 1—Temperature increase—(In the temperature range 25–79 °C): Ramp rate: 2 °C/min, shear rate: 16 s^−1^); step 2—Holding (constant temperature of 79 °C): holding time 5 min, shear rate: 10 s^−1^) and (iii) step 3—Temperature decrease (in the temperature range 79–25 °C), ramp rate: 2 °C/min, shear rate: 0 s^−1^.

Thermal and HHP-treated samples were stored at 25 °C before further analyses. All experiments were done in triplicate.

#### 2.2.3. Optical Measurements

Samples were observed using an optical inverted microscope Nikon Eclipse (TE 2000S, Nikon instruments Europe B.V., Amsterdam, The Netherlands) equipped with a polarization filter, with a 10×, 20× and 40× objective, coupled to a DS Camera Control Unit (DS-5M-L1, Nikon Instruments Europe B.V, Amsterdam, The Netherlands) for image acquisition and analysis. Before the observation, a small amount of sample was spotted on a microscope slide and covered with a cover glass. For the visual observation of starch microstructure, the light microscope with the photographing option in the DS Camera Control Unit was used.

The determination of granules optical size was performed from the images on the screen of the DS Camera Control Unit and utilizing the Equation (1), developed to determine the perimeter of the ellipse, fitting potato starch granules shape.

(1) $$p\approx \sqrt[{2\pi }]{\frac{a^{2}+b^{2}}{2}},$$

where: *p* is the approximated perimeter of an ellipse, *a* is the radius of the major axis and *b* the radius of the minor axis.

The degree of gelatinization of samples was detected measuring the loss of the optical birefringence of starch granules under polarized light (20×), calculated according to the Equation (2), developed by Buckow et al. [34].

(2) $$DG=\left(1-\frac{NB}{N}\right)\;\times \;100,$$

where: *NB* is the number of granules with birefringence and *N* the number of total granules.

The percentage of swelling of the granules in all samples was calculated according to Equation (3):

(3) $$\mathrm{Swelling}\;\mathrm{ratio}\;\mathrm{increment}\;\left(\%\right)=\;\frac{\mathrm{Initial}\;\mathrm{Size}\;\left(µm\right)-\mathrm{Size}\;\mathrm{after}\;\mathrm{HHP}\;\mathrm{treatment}\;\left(µm\right)\;}{\mathrm{Initial}\;\mathrm{size}\;\left(µm\right)}\times 100.$$

For all the optical analyses, a statistically sufficient number of starch granules were considered (225).

Water holding capacity (WHC) on the samples was determined according to Equation (4):

(4) $$\mathrm{WHC}\;\left(\%\right)=\;\frac{\mathrm{Weight}\;\mathrm{of}\;\mathrm{structured}\;\mathrm{part}\;\left(g\right)-\;\mathrm{Weight}\;\mathrm{of}\;\mathrm{dry}\;\mathrm{starch}\;\left(g\right)}{\mathrm{Weight}\;\mathrm{of}\;\mathrm{dry}\;\mathrm{starch}\;\left(g\right)}\times 100.$$

The % of the structured part in the samples was calculated using Equation (5):

(5) $$\mathrm{Structured}\;\mathrm{part}\;\left(\%\right)=\;\frac{\mathrm{Weight}\;\mathrm{of}\;\mathrm{structured}\;\mathrm{part}\;\left(g\right)\;}{\mathrm{Total}\;\mathrm{weight}\;\mathrm{of}\;\mathrm{sample}\;\left(g\right)}\times 100.$$

#### 2.2.4. Rheological Measurements

Rheological determinations of the samples were carried out in a controlled stress and strain rheometer (AR 2000, TA instruments, New Castle, DE, USA), thermally regulated by a Peltier plate and a circulating water bath (DC10-Haake K10, Karlsruhe, Germany). The instrument was fitted with a plate-cone geometry (40 mm diameter, 2°) with a fixed gap of 52 µm. For the analysis, 1.0 g of the sample was put on the center of the rheometer plate and kept at fixed measurement temperature (25 °C) for 2 min to allow stress relaxation and temperature equilibration. The experiments were carried out in triplicate. A new sample was used for each determination.

Different types of tests were carried out to determine the rheological behavior of the samples, as described in detail in the following sections. The same geometry described above was used in all the measurements.

##### Flow Measurement Tests

Steady-state flow tests were carried out in all the samples at 25 °C. The apparent viscosity (η) and shear stress (γ) of the samples were determined in the range of shear rates between 0.1 and 100 s^−1^. The equilibration time of the samples was set at 120 s.

##### Frequency Tests

Mechanical properties were obtained from frequency sweep tests recorded at 25 °C. A continuous oscillation at fixed controlled strain (3%) was utilized. The amplitude of deformation was kept constant while the frequency was changed in a selected range (0.1 to 100 rad/s). The equilibration time of the samples was set at 120 s. The storage modulus G’ and loss modulus G” as a function of frequency of the samples were recorded.

#### 2.2.5. Fourier Transform Infrared Spectroscopy (FT-IR) Measurements

The FTIR spectra of the samples were recorded with an FT/IR-400 spectrometer (Jasco Corporation, Kyoto, Japan). Of samples, 0.3 mL was spotted at the center of an IR transparent ZnSe optical disc. For each measurement, 128 scans were collected at 4 cm^−1^ resolution. The FTIR spectra of the samples were determined at wavelengths ranging from 1200–950 cm^−1^. Three samples from each experimental condition were used for each spectra measurement. The resultant starch-averaged spectrum was smoothed with a fifteen-point under adaptive-smoothing function to remove the possible noises, and then, baseline modification and normalized function were applied.

#### 2.2.6. Thermal Properties

The thermal properties of native and HHP-treated starch samples were evaluated in a differential scanning calorimeter (DSC) instrument (DSC 204 Phoenix, Netzsch, Wittelsbacherstraße, Germany). Prior to experiments, DSC was calibrated for temperature and enthalpy using indium as a standard (*T*_m_: 156.6 °C and Δ*H*_m_: 28.45 J/g). For the analysis, 20 ± 0.01 mg of HHP-treated samples were placed in a 25 µL aluminum pan. Pans were hermetically sealed, and an empty pan was used as a reference. DSC measurements were carried out through an isothermal phase (25 °C for 3 min) and then scanned at a dynamic phase at 5 °C/min from 25 to 90 °C. Denaturation temperature (T_d_) and denaturation enthalpy (Δ*H*_d_) were estimated by measuring the area under the DSC transition curve with the manufacturer software Proteus Analysis Software (Version 4.2/3, Netzsch, Wittelsbacherstraße, Germany). All the DSC measurements were done in triplicate.

% of gelatinization was calculated using Equation (6) reported by Blaszczak et al. [51]:

(6) $$\%\;Gel=\frac{\Delta \mathrm{Hns}-\;\Delta \mathrm{Hts}}{\Delta \mathrm{Hns}}\;\times \;100,$$

where Δ*H*_ns_ and Δ*H*_ts_ were the gelatinization enthalpies of native and HHP-treated potato starch, respectively.

### 2.3. Statistical Analysis

Results were analyzed by statistical descriptive analysis (mean ± SD), one-way ANOVA and posthoc comparison using the Fisher least significant difference (LSD) test to determine significant differences among experiments (*p* < 0.05). All analyses were performed using Statgraphics Centurion XVI Statistical Software (Statistical Graphics Corp., Herdon, VA, USA).

## 3. Results and Discussion

### 3.1. Effects of Starch–Water Concentration on Gel Formation and Structural Properties of Potato Starch Suspensions Treated by HHP

The results of gel formation of native and pressurized potato starch suspensions with different starch–water concentrations are reported in Table 1. Starch suspensions with concentrations in the range 10–30% (w/w) were used to investigate gel formation under high pressure conditions according to the literature [32,45,46]. At the HHP treatment conditions utilized in this work, namely 600 MPa for 15 min, a slight increase of particle size was observed, due to starch granules hydration as well as the partial gelatinization of starch suspensions. However, these results were independent of starch concentration (*p* > 0.05; Table 1). This is in agreement with experimental findings reported previously in the literature, underlining the baroresistance of potato starch under certain pressure conditions due to the chemical structure of this material (B-type starch) [39,40,41,43,48,52].

The independence of the extent of gelatinization on starch concentration observed in this study was slightly in agreement with the results reported by Kawai et al. [32,45,46]. The authors reported that with increasing the water contents of the starch suspension the degree of gelatinization was increasing. However, it should be emphasized that the processing conditions utilized in their work were by far more severe (1000 MPa for 1 h to 66 h and 40 °C). In our case, it could be assumed that a holding time of 15 min at a pressure level of 600 MPa was not sufficient to produce statistically significant differences among the potato starch suspensions at different water concentrations as far as % of gelatinization is concerned. Although the visual observation of samples could allow concluding that at lower starch concentrations more extensive gelation was occurring, however, analyzing the standard deviations there is no evidence of inhomogeneity among all partially gelatinized samples. Water holding capacity of starch granules (WHC) and % of the structured part in the sample could be considered as an indirect measurement of the amount of structured material formed under pressure. Analyzing the data reported in Table 1 it could be concluded that water holding capacity of granules (WHC) and percentage of gel-structure of samples with 20% (w/w) starch–water concentration were higher (*p* < 0.05), demonstrating that suspensions with this starch–water content were more prone to gelation at the processing conditions tested.

The effects of starch concentrations on structural properties of samples were evaluated also by FTIR. Fourier transform infrared spectroscopy (FTIR) is a vibrational technique, which provides useful information on the conformational structure of food components [53] and starch gel structures characterization [30,54,55,56,57].

**Figure 1:**
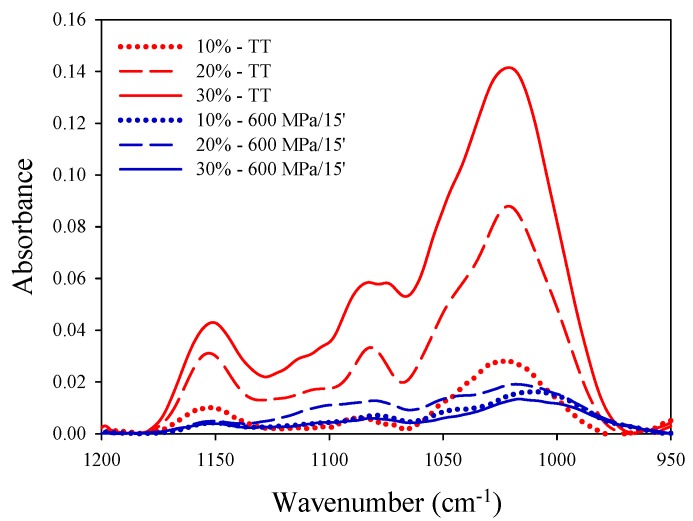
FT-IR spectra of potato starch suspensions at different starch concentration (10–30% w/w) treated by high pressure (600 MPa for 15 min at 25 °C) and thermal treatments.

In Figure 1 FTIR spectra of HHP-treated samples with different starch concentrations (10–30%) were reported. Spectra of thermal gels (control) with the same starch concentrations were also shown (red lines) as a reference (pattern of well-structured gels). In the wavelength range 950–1200 cm^−1^, it is possible to identify bands characteristic of starch gelation [50]. According to Rubens et al. [50], the bands of the infrared spectra in this range of wavelengths mainly account for C–O stretching of the ring and linkage of (C–O–C) and COH groups. Spectra variations due to pressure gelation can be determined individuating changes of intensity, changes of bandwidth and frequency shifts, being the change of intensity, the main effect occurring during gelation. In this study, the changes of intensity of peaks in the sensitive starch region (wavelength range 950–1200 cm^−1^) were determined. 

According to data reported in Figure 1, all spectra showed the characteristic absorption peaks at 1022, 1080 and 1150 cm^−1^ [56], related to the absorption bands of amorphous and crystalline regions and to the amount of ordered structures [30,56,57]. These absorptions peaks have been identified as indicators of starch gelation [58] among which the amplitude of the peak at 1022 cm^−1^ accounts for amorphous structures [54]. Thermal gels (control) showed high absorption peaks in the considered range, highlighting highly structured profiles, regardless of the starch concentration. In the starch sensitive region (Figure 1) higher absorptions, corresponding to higher starch concentrations, were evidenced. Similar results were reported by Van Soest et al. [30] in a study on the influence of water content on the deconvoluted spectra of potato starch solutions at different water concentrations. The authors demonstrated that with increasing water concentration an increased intensity of the peak at 1022 cm^−1^ could be detected (overlapping the band at 994 cm^−1^).

Pressurized potato starch samples showed lower absorbance values in the sensitive region (950–1200 cm^−1^) as a result of the partial formation of a gel structure. Slightly higher absorption values of the amorphous and ordered structures were detected in samples with 20% (w/w) starch content with respect to samples with different starch concentrations. This demonstrated that HHP treatment of starch suspensions at 20% (w/w) starch–water concentration was more effective and allowed the formation of samples with a more structured profile. Although through FTIR spectra the influence of water concentration on the structural behavior of HHP-treated starch solutions could not be assessed, the results of FTIR analyses enabled highlighting the extent of gelation and as such, they were in good agreement with the results of optical and physical parameters measurements of both untreated and HHP-treated starch suspensions. 

Further analyses described in the following, were carried out on samples with 20% (w/w) starch–water concentration.

### 3.2. Effects of Particle Size Distribution on Gel Formation and Structural Properties of Potato Starch Suspensions Treated by HHP

Based on previous results, starch gelatinization by HHP depended on granules particle size (data not shown). It is well known that the gelatinization of starches with small granules size, such as rice, wheat, corn and pea starch, takes place more easily at the processing conditions utilized in this investigation, namely 600 MPa for 15 min [40,43]. However, as already discussed in Section 3.1, potato starch is characterized by a significant pressure resistance to gelatinization, which has been mainly attributed by other authors to its chemical spatial conformation (B-type) without considering other possible influencing factors, such as granules size. To confirm this hypothesis, the effects of particle size distribution on structural properties of potato starch suspensions and on gel formation were evaluated. 

The results of gel formation of native and pressurized potato starch samples with different mean particle size are reported in Table 2. 

Data reported in Table 2 clearly showed that with smaller starch granules (<25 µm) gel formation at HHP treatment conditions investigated was facilitated (*p* < 0.05). In particular, considering the swelling ratio, which is the difference between starch granules size before and after HHP treatment, it was possible to observe an increase of size of 74.6% for small granules (<25 µm) while this increase was 14.9% and 22.8% only for medium granules size (50–63 µm) and for the unsieved starch granules, respectively. Although to the best of our knowledge these findings were never reported in the literature for potato suspensions treated by HHP, this behavior could be explained taking into account that small particles move during HHP treatment and they are more exposed to the action of pressure than bigger particles. Moreover, being the number of particles per unit volume increased when the particle size decreases, starch–starch and starch–water interactions in suspensions of small starch granules are much higher in the space available, thus a stronger network can be formed. Ahmed (2018) reported that a higher effect of HHP-treatments (>450 MPa) was detected on tapioca starch suspensions of small granules size rather than on those of bigger size. At 600 MPa, a size increment of 329% of the smaller granules size portion Dv_10_ (from 1.56 µm to 6.69 µm) was detected, while an increase of only 55% and 15% were detected on the granule size portion (Dv50) and on the overall granule size distribution (Dv90). The authors explained these findings considering the ability of small granules to binding more water, due to the higher surface area which may lead to a more efficient hydration and, consequently, a higher swelling [59]. Similar observations were reported by other authors on barley, wheat and potato starch suspensions gelatinized by thermal treatments [60,61,62].

**Figure 2:**
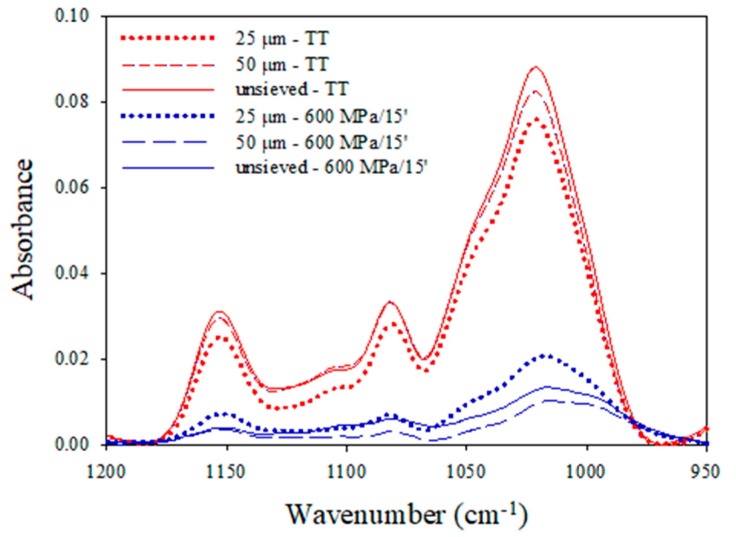
FT-IR spectra of potato starch suspensions (20% w/w) of different particle sizes treated by high pressure (600 MPa for 15 min at 25 °C) and thermal treatments.

In Figure 2 FTIR spectra of thermal and pressurized potato starch samples of different mean particle size are reported. Thermal gels showed a characteristic spectrum of well-structured gels, being the absorption peaks in the region of interest more pronounced with increasing the size of the granules. Samples obtained at 25 °C after pressurization at 600 MPa for 15 min showed an opposite trend. When granules of smaller size (<25 µm) were present in the suspension better structure profiles were observed while poorer structured samples were obtained with bigger starch granules size (36–50 µm) and unsieved potato starch, confirming that the smaller the granules size the higher the effectiveness of HHP treatments on starch gelation, thus reinforcing the results of Table 2. Observing the absorption peak at 1022 cm^−1^, which, according to Warren et al. [54], is influenced by the water content of the starch suspension, the influence of particle size distribution clearly appeared on both thermal and pressurized samples. Thus, it can be concluded that the utilization of starch with small size positively affects potato starch gelation by HHP as well as the structural properties of the samples obtained. Further experiments presented in the following have been carried out on suspensions of small granules size (<25 µm) at 20% (w/w) starch concentration.

### 3.3. Effects of Combined HHP-Thermal Treatments on Gel Formation, Structural and Mechanical Properties of Potato Starch Suspensions

According to the results presented so far, at the HHP treatment conditions investigated, namely 600 MPa for 15 at 25 °C, it was not possible to induce the complete gelatinization on potato the starch suspensions utilized. Thus, other experiments were carried out at higher processing temperatures (40 °C and 50 °C) to evaluate the influence of moderate heating on HHP gel formation, structural and mechanical properties of products, in a hurdle approach.

**Figure 3:**
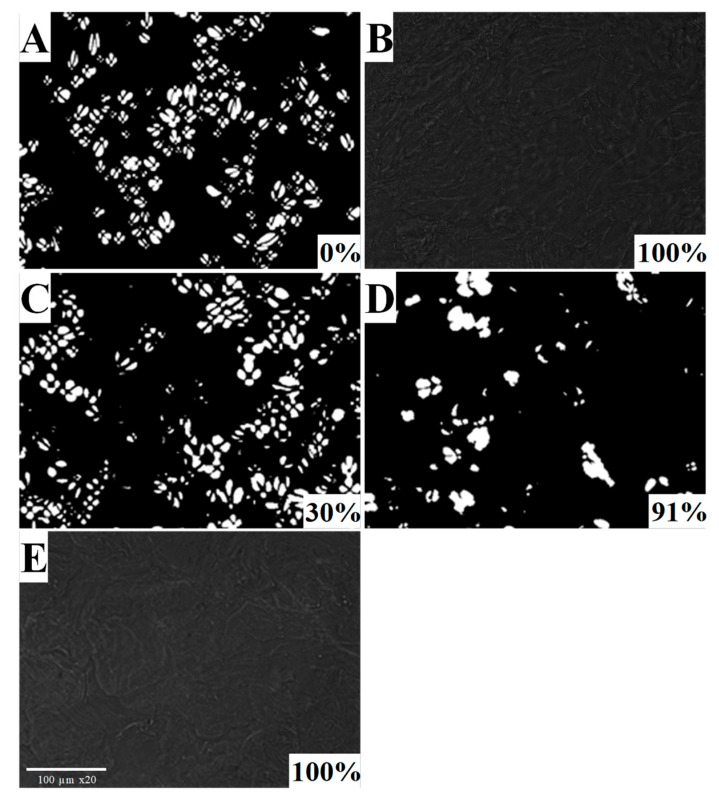
Polarized light micrographs of untreated potato starch suspensions (25 µm, 20% w/w) (**A**), thermal treated (**B**), pressure treated at 600 MPa for 15 min at different processing temperatures, namely 25 °C (**C**), 40 °C (**D**) and 50 °C (**E**), respectively. The % of gelatinization of samples, determined as a function of birefringence loss, are also indicated in the pictures.

Potato starch suspensions (starch concentration of 20% (w/w) and granules size <25 µm) were treated at 600 MPa for 15 min at 40 °C and 50 °C. The changes of birefringence of the treated samples were reported in Figure 3. Under polarized light, native starch granules show birefringence in the typical form of a “maltese cross” [36]. Normal birefringence and no-birefringence were clearly observed on untreated and thermal treated potato starch suspensions, respectively. These patterns were used for comparison (Figure 3a,b). Data from Figure 3 demonstrated that the loss of birefringence was increasing with increasing the treatment temperature. Birefringence of potato starch granules was reduced after HHP treatments at 25 °C (Figure 3c), while it was almost completely lost after treatment at 40 °C (91%; Figure 3d). Furthermore, potato starch suspensions HHP treated at 600 MPa for 15 min at 50 °C showed a complete loss of birefringence (Figure 3e), accounting for a complete gelatinization and hydrogel formation. Our results were in good agreement with those of other authors [32,35,45,46]. Although many research efforts have been made to study HHP potato starch gelatinization, only a few papers reported results on gel formation at economically feasible processing conditions [35,40,41,48,50,63]. Bauer and Knorr [39] reported that HHP induced the complete gelatinization of potato starch suspensions (5% w/w) at 700 MPa for 15 min and 50 °C, and Kawai et al. [32,45,46] reported that the complete gelation of potato starch suspensions (20% w/w) occurred at 800 MPa and 40 °C for processing time longer than 60 min. This study demonstrated that the complete gelatinization of potato starch suspension could be obtained at lower pressure levels, provided that a combination of pressure and moderate heating treatment (at 50 °C) was applied. With increasing the treatment temperature, the molecular motions are favored, and the simultaneous application of heat and pressure had a synergetic effect on the gelatinization process [32].

**Figure 4:**
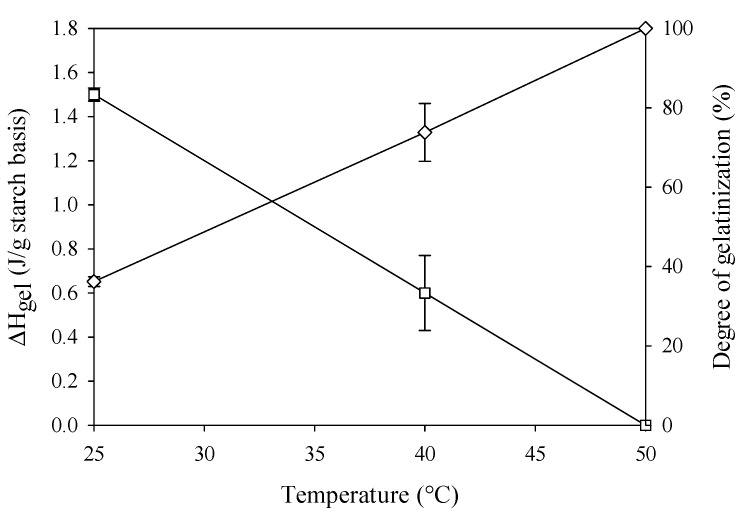
ΔH_gel_ (J/g starch, open square) and degree of gelatinization (%, open diamond) as a function of HHP processing temperatures of potato starch suspensions (25 µm, 20% w/w) treated at 600 MPa for 15 min.

In order to obtain information on the degree of gelatinization of potato starch suspensions obtained at the above-mentioned processing conditions, thermal analysis by DSC was performed. Gelatinization enthalpy (Δ*H*_gel_) and degree of gelatinization (*D*_g_) of each HHP treated sample are shown in Figure 4. According to data of Figure 4, Δ*H*_gel_ decreases with increasing the processing temperature. Consequently, the increase of *D*_g_ measured confirmed the results of microscopy tests (Figure 3). However, higher *D*_g_ values shown in Figure 4 allowed us concluding that the birefringence loss method underestimates the extent of gelatinization as already discussed by Kawai et al. [32]. The authors hypothesized that very small granules of potato starch can keep their birefringence, thus the underestimation of the degree of gelatinization through microscopy technique is likely to occur. Although the determination of the degree of gelatinization is strongly dependent on the testing method, the aim of this study was to confirm that HHP induces the complete gelatinization of potato starch suspensions at 50 °C, which was clearly confirmed by both methods utilized.

**Figure 5:**
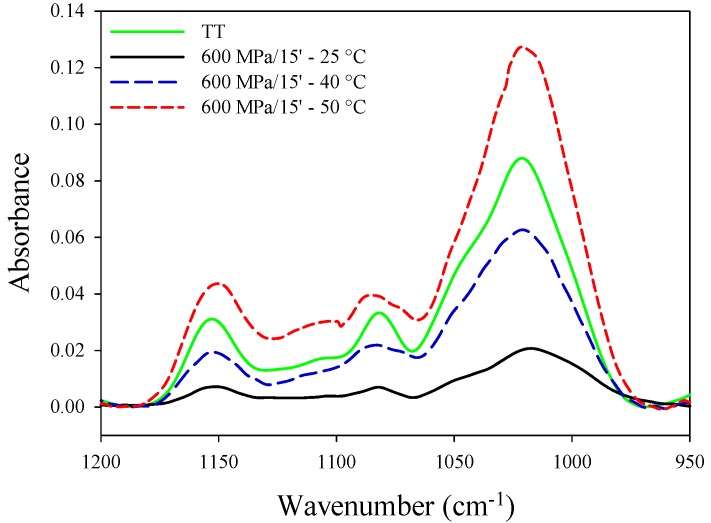
FT-IR spectra of potato starch suspension (20% w/w) treated by HHP at different processing temperatures.

Data reported in Figure 5 demonstrated the effects of processing temperatures (25 °C, 40 °C and 50 °C) on the structural properties of HHP hydrogels. The spectrum of a thermal gel, representing a highly structured material, was also depicted in Figure 5.

The intensity of all peaks in the sensitive region (950–1200 cm^−1^) tended to increase with increasing the processing temperature. According to van Soest et al. [30], alterations in this wavelength region are expected as gelation causes damages in the crystalline region and, consequently, the increase of the amorphous structures. HHP treated samples at the processing temperature of 50 °C showed the highest absorption peaks in the sensitive region, accounting for a highly structured profile, even higher than the spectrum of thermal gel (TT). It could be concluded that combined high pressure and thermal treatments produced a synergistic effect on hydrogels structure, resulting in stronger and more dense networks due to strong inter and intra-molecular interactions, clearly highlighted in FTIR spectra.

**Figure 6:**
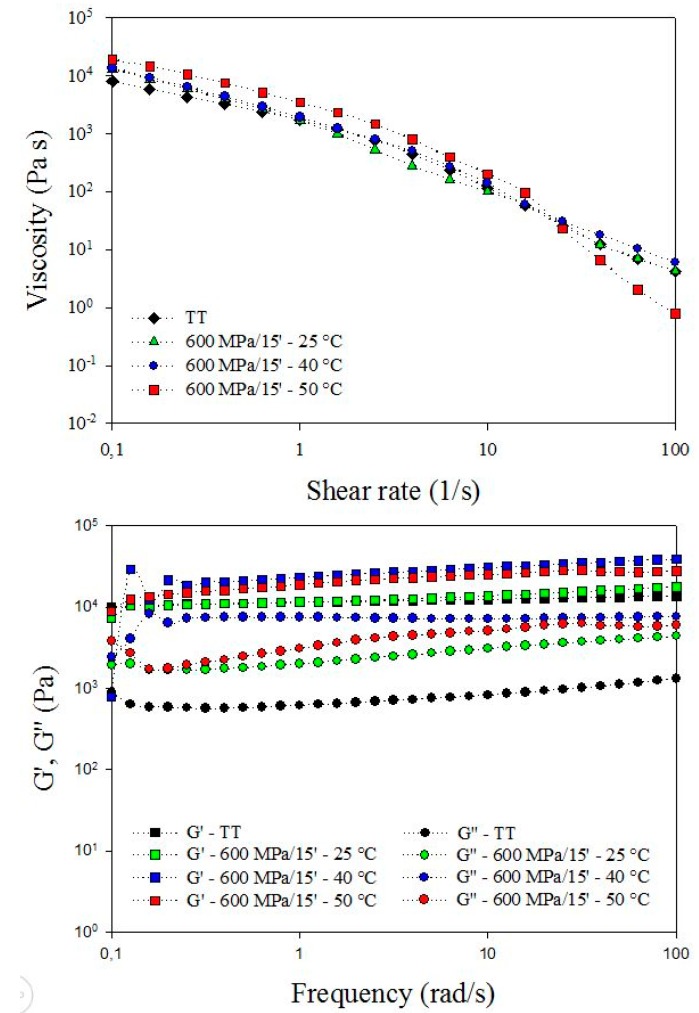
Rheology of potato starch suspension (25 µm, 20% w/w) subjected to thermal and high pressure treatments at 600 MPa for 15 min at 25 °C, 40 °C and 50 °C.

The effects of processing conditions on mechanical properties of hydrogels were assessed by rheological measurements. Flow curves and viscoelastic properties of thermal and HPP hydrogels (600 MPa for 15 min at 25 °C, 40 °C and 50 °C) are shown in Figure 6. According to the trend of the flow curves in the stationary regime, all samples exhibited a non-Newtonian shear-thinning behavior, independently on processing conditions. Such rheological behavior is typical of starch gels, as reported in the literature [29,64]. Generally, starch gels are irreversibly broken under shear forces, involving a reorganization of the molecules and the reduction of the intermolecular resistance to flow and, thus, they show a shear-dependent flow behavior [65,66]. 

**Figure 7:**
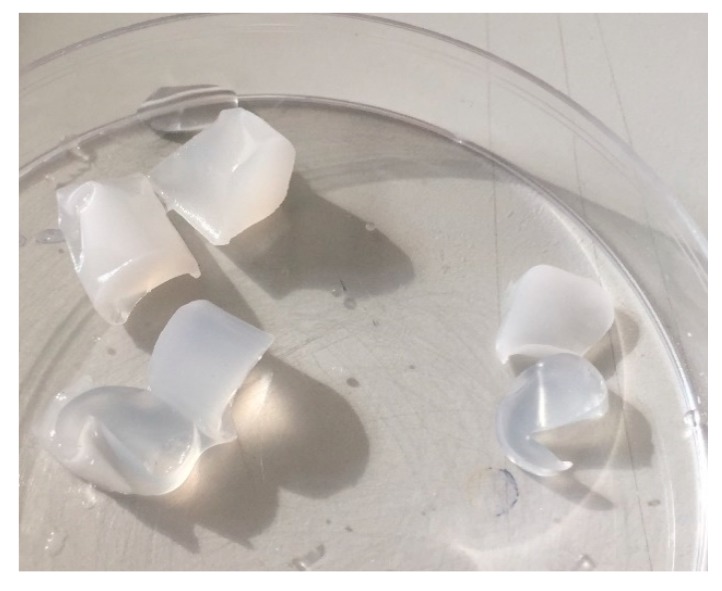
Picture of the potato starch hydrogels obtained in this investigation at fixed conditions (starch-water concentration of 20% w/w, mean size of <25 µm and processed at 600 MPa for 15 min at 50 °C).

Despite their shear-thinning behavior, slight differences among samples obtained at the different processing conditions investigated were detected. The initial and final viscosity of thermal and HHP hydrogels obtained at 25 °C and 40 °C are similar in the shear rates range 0.1–100 s^−1^. At lower shear rates (0.1 to 15 s^−1^), the viscosity of hydrogels obtained at 600 MPa for 15 min and at 50 °C was higher. However, at higher shear rates (>15 s^−1^) the viscosity of HHP hydrogels decreased abruptly with respect to that of samples obtained at a lower temperature. This behavior could be attributed to the more structured and compact network formed by HHP treatment at 50 °C (Figure 5 and Figure 7), which is fractured under higher shear forces, as demonstrated by the lower final viscosity values.

The mechanical spectrum, also known as the frequency sweep test, is also utilized to classify products, materials or gels [67,68,69]. Standard rheological characterization of the viscoelastic properties of complex fluids and gels is traditionally carried out in the frequency domain where gels are subjected to deformation forces and the stress response is measured [70]. The mechanical behavior of samples was obtained as a function of G’ (elastic) and G” (viscous) moduli in the frequency sweep range 0.1–100 (rad/s) at 25 °C. Viscoelastic properties of HHP-treated samples at different temperatures (25 °C, 40 °C and 50 °C) are reported in Figure 6. Thermal gel (control) curves were also added for comparison. Frequency sweep curves of all samples showed the typical trend of gel structures, with G’ greater than G’’, indicating a predominance of elastic over viscous properties [68,69]. Moreover, G’ and G’’ values of fully gelatinized samples (600 MPa for 15 at 50 °C and control) were higher than those of partially gelatinized samples (600 MPa for 15 at 25 °C and 40 °C), in agreement with the conclusions reported in a recent paper on fully HHP-gelatinized and not gelatinized quinoa starch [59].

One of the main objectives of determining the viscoelastic properties of gels by frequency sweep tests was evaluating the strength of the gels, which is a function of the position of G’ and G’’ curves [65], and the dependence of G’ and G’’ in the frequency range considered [71]. The position of G’ and G’’ curves reported in Figure 6 clearly indicated that fully gelatinized samples showed mechanical characteristics typical of strong gels. Moreover, hydrogels formed by the synergistic high pressure-thermal treatment at moderate temperature (600 MPa for 15 min at 50 °C) showed a weak frequency-dependence, which indicated a superior gel strength profile. According to Douglas [70], “strong gels” are characterized by an elastic response to shear deformations due to the well-structured network that stores the energy when stress forces are applied to the material. HHP-thermal hydrogels obtained at 600 MPa for 15 min at 50 °C showed the characteristics of strong gels described above, as also confirmed by the other properties measured, namely flow behavior and structural properties.

## 4. Conclusions

Although in the literature several papers described that HHP is a suitable technology to obtain potato starch hydrogels, starting from suspensions at different starch concentrations, and illustrated the most appropriate processing conditions, to the best of our knowledge no data have been published on the characterization of the products obtained. In this study, the effects of starch concentration in the suspension (10–30% w/w), particle size distribution (<25 µm, 50 µm and unsieved) and HHP processing temperature (25, 40 and 50 °C) on potato starch gel formation were studied and structural and mechanical properties of the products obtained were investigated.

The analysis of the results allowed concluding that high hydrostatic pressure was effective to produce potato starch-based hydrogels at economically feasible conditions, with processing times significantly lower than those used in conventional gelation methods. At a starch concentration of 20% w/w, the utilization of granules with small size (<25 µm) positively affected gel formation by high pressure, and a hurdle approach was necessary, that is coupling high pressure with moderate heating at low temperature (50 °C), to obtain a stable hydrogel with excellent structural and mechanical properties, even superior to those of thermal gels.

More work is needed to further characterize these hydrogels through measurements of the stability of these structures, the determination of the optimal storage conditions as well as the investigation of the effects of adding others compounds on product properties in view of their applications in different industrial sectors.

## Figures and Tables

**Table 1:** Optical and physical parameters of untreated and high hydrostatic pressure (HHP) treated (600 MPa for 15 min at 25 °C) potato starch suspensions at different starch concentration.

	Starch–Water Concentration		
	10% w/w	20% w/w	30% w/w
**Samples**	**Average Granule Size (µm)**		
**Native**	50.9 ± 24.4 ^a^		
**HHP-treated**	59.9 ± 32.4 ^Ab^	62.5 ± 20.9 ^Ab^	56.7 ± 27.1 ^Aa^
**HHP-treated**	**Swelling %**		
	17.7	22.8	11.4
	**% of Gelatinization**		
	18.5 ± 8.2 ^A^	16.6 ± 7.5 ^A^	12.6 ± 6.9 ^A^
	**WHC**		
	70.1 ± 2.3 ^A^	71.2 ± 2.6 ^A^	48.2 ± 6.3 ^B^
	**% of Structured Part in the Sample**		
	33.5 ± 2.6 ^B^	69.9 ± 6.3 ^A^	58.3 ± 7.1 ^A^

All values are means of 225 (granules) determinations ± SD. ^(a,b)^ Values in the same row with different superscripts differ significantly (*p* < 0.05; least significant difference—LSD). ^(A,B)^ Values in the same column with different superscripts differ significantly (*p* < 0.05; LSD).

**Table 2:** Swelling behavior and % of gelation of potato starch suspensions (20% w/w) of different particle size, after pressure (600 MPa for 15 min at 25 °C) and thermal treatments, determined from optical measurements.

	Mean Size		
	<25 µm	50 µm	Unsieved
**Samples**	**Average Granule Size (µm)**		
**Native**	28.0 ± 8.6 ^Cb^	70.0 ± 6.3 ^Ab^	50.9 ± 24.4 ^Bb^
**HHP-treated**	48.9 ± 14.0 ^Ca^	80.4 ± 13.2 ^Aa^	62.5 ± 20.9 ^Ba^
**HHP-treated**	**Swelling %**		
	74.6	14.9	22.8
	**% of Gelatinization**		
	30.2 ± 4.3 ^A^	13.8 ± 0.8 ^B^	16.6 ± 7.5 ^B^

All values are means of 225 (granules) determinations ± SD. ^(A,C)^ Values in the same row with different superscripts differ significantly (*p* < 0.05; LSD). ^(a,c)^ Values in the same column with different superscripts differ significantly (*p* < 0.05; LSD).
